# Supplementary material for: Determinants of in-hospital mortality in COVID-19; a prospective cohort study from Pakistan
Source: PLoS One. 2021 May 27;16(5):e0251754. doi: 10.1371/journal.pone.0251754 (PMC8158897; doi:10.1371/journal.pone.0251754)
Supplement: Attachment — Submitted filename: Response to reviewers.docx [file pone.0251754.s001.docx]

Dear Editor and respected reviewers,

PLOS ONE,

I am writing to you with reference to our submission titled “Determinants of in-hospital mortality in COVID-19; a prospective cohort study from Pakistan” (PNOE-D-21-00091). On behalf of my co-authors, I would like to thank the journal and respected reviewers for their time and effort in providing us with a comprehensive review highlighting the issues with the manuscript. We are indebted to the journal for providing us with a second chance at the submission.

In the light of the reviewers’ suggestions, the analysis was redone and results written again. Below we provide a point-by-point reply to the reviewers on their concerns. We hope to satisfy the journal and reviewers with these modifications in the revised version.

Dr Quratulain Shaikh

Corresponding author

Reviewers' comments:

Reviewer's Responses to Questions

**Comments to the Author**

1. Is the manuscript technically sound, and do the data support the conclusions?

The manuscript must describe a technically sound piece of scientific research with data that supports the conclusions. Experiments must have been conducted rigorously, with appropriate controls, replication, and sample sizes. The conclusions must be drawn appropriately based on the data presented.

Reviewer #1: No

Reviewer #2: Partly

Reviewer #3: Partly

2. Has the statistical analysis been performed appropriately and rigorously?

Reviewer #1: No

Reviewer #2: Yes

Reviewer #3: No

3. Have the authors made all data underlying the findings in their manuscript fully available?

The [PLOS Data policy](http://www.plosone.org/static/policies.action#sharing) requires authors to make all data underlying the findings described in their manuscript fully available without restriction, with rare exception (please refer to the Data Availability Statement in the manuscript PDF file). The data should be provided as part of the manuscript or its supporting information, or deposited to a public repository. For example, in addition to summary statistics, the data points behind means, medians and variance measures should be available. If there are restrictions on publicly sharing data—e.g. participant privacy or use of data from a third party—those must be specified.

Reviewer #1: No

Reviewer #2: No

Reviewer #3: Yes

4. Is the manuscript presented in an intelligible fashion and written in standard English?

PLOS ONE does not copyedit accepted manuscripts, so the language in submitted articles must be clear, correct, and unambiguous. Any typographical or grammatical errors should be corrected at revision, so please note any specific errors here.

Reviewer #1: Yes

Reviewer #2: Yes

Reviewer #3: Yes

5. Review Comments to the Author

Please use the space provided to explain your answers to the questions above. You may also include additional comments for the author, including concerns about dual publication, research ethics, or publication ethics. (Please upload your review as an attachment if it exceeds 20,000 characters)

Reviewer #1: The paper is well writtem and as noted by the authors in the introduction, these data could help in risk stratification and management of COVID-19 patients. The main issue of the paper resides in the fact that the treatments assignments are not randomized so that any conclusion regarding the association between treatment and survival is dubious. There are also issues in the presentation of the statistical analysis that should be (easily) corrected.

I would recommend to the authors to state clearly the assumptions they want to investigate and a major revision of the statistical analysis before any resubmission.

Acknowledged


Statistical aspects
-------------------
It is not clear whether the study followed a pre-speficied statistical analysis plan. For example, was the criteria that led to the exclusion of patients under 15 set before the start of the data analysis? If so, were the various pre-defined steps registered anywhere?

The study followed a pre-specified statistical analysis plan which was mentioned in the IRB approved proposal. However, it was not published or registered in any repository. Patient’s younger than 18 years were excluded (15 was a typographical error) as our inclusion criteria included adult patient 18 and above. The unit had pediatric as well as adult admissions but pediatric cases were few in number.

p11 l 99 "Among the rest 384 (9.9%) needed admission but due to limited bed availability in the isolation unit, only 193 (50%) were admitted while the rest were referred to other isolation units in the city."
What was the rationale underlying the choice of patients referred to other isolation units?

Bed availability in the isolation unit was the only criteria based on chronological order of request for admission. If the bed was available then patient was admitted and if there was no vacant bed then they were referred for admission to other centers through a central database displaying real time availability of isolation beds created by the government of Pakistan for facilitating serious COVID patients

Also it appears later in the text and table 1 that some of the 186 patients admitted were asymptomatic (n=2) or presenting mild symptoms (n=13). Please expain.

Some COVID positive patients did not need admission for management of COVID but needed inpatient management of other disease – there were 16 such patients who needed dialysis, or were admitted due to surgical indications hence were included in the analysis. However, after reading the reviewer’s comments we understand the bias it may create to the results and hence the analysis has been redone after excluding them now. Total sample size= 170 after exclusion of those 16 patients

The statistical analysis reported at lines 110-142 table 1&2 is supported by various p-values. Those p-values have to be taken with a grain of salt since we are in the classical setting of multiplicity or multiple testing. With a number of hypotheses tested large enough, there will always be some significant p values by chance alone. Therefore, it would be good to report which p-values are significant after Benjamini-Hochberg correction.

We are thankful to the reviewers for highlighting this. We have now removed the p-values from the tables and only describing the data for survivors and non-survivors in Table 1 and 2. However, if the reviewers think that p-values should be reported then we can perform the Benjamini-Hochberg correction and redo these tables and results.


Table 3 and l 144-158 the assignment of treatment being non randomized, it is difficult to interpet any association between treatment and outcome.
Besides, table 3 report various frequencies of treatment conditionnal on survival status. Even in case of an RCT, such numbers would be difficult to interpret. What is required indeed is a frequency of survival conditionnal on treatment.
For those two reasons, a conclusion like l. 155 "There was no mortality benefit of using methylprednisolone in our patients (p>0.05)" is not grounded at all.
This table should be left as is and would stand as a purely descriptive report of the pathways of cares.
A table of frequency of survival conditional on treatment should be provided, but again, any attempt to draw conclusion about the observed association should be avoided.

Agreed with the comments. We have removed inferential analysis from this table in the light of suggestions from reviewers. Please let us know if the respected reviewers would like to see the table otherwise. Thanks


l 160 Please explain better what is meant by "Univariable binary logistic regression (Table 4) at a cut-off of p<0.10, showed that ...".
Do you mean that you implemented a logistic regression for all variables and present here only the results for variables with the lowest p-values?

A cut off of <0.1 is mentioned here as significance was pre decided at <0.1 for a univariable binary regression. However, since now the analysis has been redone, the selection of variables for multivariable model building was not only based on statistical criteria but alos based on available literature.

Table 4: this table mixes variables of different types: demographic parameters, clinical characteristics at admission, baseline lab parameters and treatments. The treatment being non-randomized, reporting and commenting a p-value for treatment variables makes little sense. Please also add a column with the significance obtained by the Bonferroni-Hochberg procedure.

l 170-175: can you please rephrase/explain what multivariate logistic regression was implemented and what was the goal/rationale for it. Again, it seems to mix variables of different types.
I understand the idea of implementing a multivariate logistic regression to predict the survival status of of a patient at admission, but such an approach should involve not treatment variables.
What is the goal of the multivariate regression involving all the variables?

Acknowledged! The model has been reconstructed including only predictors which were available on admission

Specific comments
-----------------


p2 Funded/unfunded study statement missing-added

p4 Human Subject Research (involving human
participants and/or tissue) missing-added

p9 l52 "Our mortality rate of 2% (3) is comparable to the India (1.45%) but lower"
-> "The Pakistan mortality rate of 2% (3) is comparable to that of India (1.45%) but lower than that of Iran (4.68% )"—modified as advised

p9 l58 "The 58 peak of infection in the first wave reached in..."
-> The 58 peak of infection in the first wave was reached in... modified as advised


p10 Do I understand correctly that asymptomatic patients or with mild symtoms were included in this study?

Yes, however, they have been excluded in this re-analysis now. The reason for their inclusion in the previous analysis was that they did needed admission not due to COVID complications per se but other medical issues namely, dialysis, and surgical emergencies. However, acknowledging the bias it may introduce to the estimates (as these were non severe disease patients) we have now excluded these patients from the analysis

p10 l88 "variables were expressed as"
-> variables were summarized by-- modified as advised

p 20 l88 "Categorical variables were expressed as number (%)."
-> "Distribution of categorical variables were expressed as percentages of the various categories."-- modified as advised

p10 l89 "Student’s t-test or Mann Whitney U test were used to compare continuous data. Chi square or Fischer’s Exact test was used to compare categorical data."
This statement is a bit vague at this step

Since these tests were not performed in the re-analysis hence this statement has been removed

p10 l91 "All variables with p-value <0.10 were considered for multivariable binary logistic regression model in order of significance." I do not not understand the last bit of this sentence "in order of significance."

We apologize for the confusion but what we meant to write was that variables who tested significant (p<0.10) were considered for final model. The model was built with stepwise regression. The new model building strategy is different from previous. We have consulted a statistician who has improvised the analysis

p11 l101 "Seven patients who were admitted at TIH were less than years old hence removed from this analysis." Please explain why.

Patient’s younger than 18 years were excluded (15 was a typographical error) as our inclusion criteria included adult patient 18 and above. The unit had pediatric as well as adult admissions but pediatric cases were few in number. Since this paper was aimed to look at adult patients characteristics hence pediatric population was excluded.

p11 l113 "fatigue was common in non-survivors and vomiting was seen in survivors (p>0.05)"
It is not clear to which test this pvalue refers to. Please clarify.

Inferential analysis has been removed now. Just describing the symptoms. Thank you for highlighting

Reviewer #2: The information included in this article is of critical and immediate importance. While the sample size may be relatively small, it is only through publication of work such as this that we can build the literature to see the bigger picture. Because of its importance, and the critical eye on science in this area, it is crucial that the results be communicated effectively and scientifically.

In that vein, I would ask the authors to take a second look at the overall manuscript and clean up small errors and inconsistencies. Most importantly, the statistical interpretation of the data needs improvement. There is an over-reliance on p-values of more or less than a threshold and not nearly enough attention paid to the data itself, which is the more important interpretation. The p-value tells us only whether what we are seeing is likely to be a random result; it cannot speak to the magnitude or importance of an effect.

Acknowledged and appreciate the reviewer’s kind input. We have rewritten the results and updated the discussion after a repeat analysis of the data. We hope it will satisfy the reviewers

Introduction
- Could use another run through by the authors to tighten up language and remove small errors; but is intelligible.
- The end of the introduction needs to be more direct about what is being estimated. From Line 64, you aren’t estimating in-hospital mortality of Covid-19 in Pakistan, but in the Indus Hospital’s COVID isolation unit.

Methods
- The Methods section needs to be re-organized into paragraphs, currently the information jumps around topically from one sentence to the next making it hard to follow. Suggest:
o Line 74, put IRB approval on its own at the end
o Line 81, a description of the types of patients admitted should immediately follow Line 73 where you describe who was included in the study
o In a new paragraph, Lines 75-87 (apart from the above) describe how the patients were recorded and followed and make a single coherent paragraph
o From Line 87 to the end (“Data was recorded…”) this should be a new paragraph on the data analysis.
Results
- Line 99 – how was it decided who needed admission? What was the criteria? I think it is moderate to severe, but this should be stated more plainly. Yes, it was moderate disease and worse. Has been added to the description now. Thanks
- A brief background on the teleconsultation service and its role, could go in introduction or methods- acknowledged
- Line 99 – in Figure 1 it is indicated 9.97 needed admission, this should be rounded to 10.0 not 9.9. Corrected, thanks
- Percentages should be displayed consistently – some are rounded to whole numbers and other to 00.0%. If significant digits, that would be fine, too, but that isn’t consistent either. Even if the number is, for example, 36.0% the display should be consistent. Acknowledged. Thanks, corrections have been made to maintain consistency
- Lines 101-102: the 7 patients <18 & excluded from the analysis need to be included on Figure 1 with a final box for the included cohort for analysis.
- Figure 1 – what does LAMA mean? Apologies, it means Leave Against Medical Advice, includes patients who were not ready to get admitted despite advice from the physicians
- Table 1 (and all others) put a space between n & (%), e.g. 8 (12.1%); true also of mean (SD) and median (IQR) Acknowledged. Corrected
- Table 1 – headers of n (%) are not applicable to continuous variables – needs to be stated in the table what 61.4 (54.1-69.1) are. The use of ^ and ^^ is non-standard and not clear enough. It appears that even the authors got mixed up (see next point). Corrected. Thanks
- Table 1 / Line 103-104 – the text says that 61.4 is a median, but the table says 61.4 is a mean, using the ^^ nomenclature defined at the bottom of the table.
- Line 103-104 and 110-111 is the same sentence repeated Corrected
- Line 111 [incorrect numbers, conclusion]– the statement that males are more likely to die because there are 77.6% males in the non-survivors compared to 76.5% males in the survivors, p<0.05 is nonsensical and at odds with what is presented in Table 1 where there is a p-value of 0.859 comparing the distribution of gender in the survivors to the distribution of gender in the non-survivors.

Apologies, yes that was a misinterpretation and error at our end. Has been corrected according to the re-analysis

- Lines 112-113, what does the p>0.05 refer to? Why not put the actual p-value? But more importantly, I am unsure the authors are correctly interpreting what each p-value in Table 1 represents. In the case of the Symptoms, the p-value in Table 1 using the chi-square method only says that across ALL of the symptoms presented among the survivors and non-survivors, there is not a statistically significant difference in this distribution. It does NOT relate to which are the three most common symptoms in both groups. Also, the statement about the 3 most prevalent symptoms does not need a p-value. Removed and edited appropriately as per new results.
- Lines 114-115, again, I cannot decipher what this p-value is supposed to represent or where it came from. Corrected
- Line 118 – I find the conclusion that temperature among non-survivors was higher suspect based on the data presented and that fact that the p-value is marked by a “D” which is not defined by the authors. Also, if this finding is real, then address the fact that differences were only by a few tenths of a degree. Acknowledged. We have removed p- values from this table to avoid such observations by chance
- Tables – there are some errant “a” and “b” in the tables which appear to have no discernable meaning. Those were erroneously put in the tables. Removed now.

Not continuing line by line, the authors need to consult a statistician on the interpretation of p-values. Furthermore, their utility in this kind of paper is not very high. The authors would do better not to try to repeat all of the information in the tables in the results, but only to highlight the findings of most interest. Each sentence in the results does not need a p-value. Results such as prevalence of certain symptoms are of interest on their own and do not neatly adhere to a p-value framework. Agreed. Have rewritten the results. Hopefully, would be acceptable for the reviewers.

Table 4 - For binary variables, the data provided in prior tables is sufficient to re-run the univariable logistic regression models. Having done this for a few, there may be transcribing errors. I used R and not Stata, but for example, for Gender (male) the OR I get is 1.07 after appropriate rounding of 1.067, the 95% CI I get is 0.53 – 2.22 after appropriate rounding of 0.528 and 2.218. These differences could be due to different statistical programs being run. However, there are also inconsistent numbers of digits displayed for the 95% CIs and the p-values. Yes, this has been redone, with univariable logistic regression done only on variables which were pertinent from literature and plausible to have association with mortality. Moreover, Linearity assumptions were confirmed for continuous variables and those not fulfilling the assumption were not considered for univariable regression

Figure 2 - The depiction of multivariable logistic regression coefficients (ORs) as a forest plot is not helpful. It would be better to display this information in a table. Forest plots such as the one included are typically used to evaluate effect estimates across different studies, not within a single model.

The figure has been replaced with a table now. Appreciate the guidance.

Interpretation – the authors need to re-assess their interpretation of their OR results for both the univariable and multivariable models. All values with a significant p-value are not “associated with high odds of mortality”. A large effect size along with a significant p-value can be used to establish an association with a high odds of mortality, but a statistically significant p-value alone is not enough. More attention should be paid to the meaning of the OR and its interpretation and less to p-values. NOTE: in the abstract the phrase ‘higher odds of mortality’ is used – this is much more accurate.

Thank you for highlighting the issues with interpretation. The results and discussion have been revised according to the new analysis. Please review them and guide us if some changes are still required

Discussion
The discussion appears to be more carefully written. It focuses on important findings and does not over extend the interpretation of p-values (e.g. "The final model shows that the factors associated with mortality are…" which is an accurate interpretation). It could still go further if effect size (i.e. the value of the OR matters!) are interpreted. Thank you for the kind words. We have now elaborated the interpretation of the final model in terms of its effect size.

Can the authors address any potential bias from patients being referred to other centers? Were more severe patients retained? Or is it assured that these were random depending only on available beds at the time of entry?

This is a very important observation. We do acknowledge the bias that has been introduced at random. These limitations skipped our mind while writing the discussion. However, we are grateful that the reviewers have brought them to our attention. It has helped us improve the quality of the manuscript. However, the referral was at random depending only on bed availability

Can the authors address the reliability of the data? Was there any missing data for patients included – i.e. this being real world data could tests performed have failed to be recorded? You included many continuous laboratory values, what is the likelihood of measurement error? Measurement error is likely to reduce the observed associated with mortality. Finally, is it possible for the categorical values to have any important misclassification? As it is expected this information will be used in larger meta-analyses and further research, inclusion of any such limitations may be invaluable.

Yes of course! Measurement errors along with misclassification are both potential biases in this study. We have added those in our discussion.

Abstract
The abstract is also over-reliant on p-value thresholds. It also says that those who died were more likely to be males, implying that they were more likely to be males than those who did not die – which is not supported by the data (see above).

Abstract has been re-written. Thank you for the suggestions and identifying the error

Reviewer #3: I appreciate the intent of this research and the need for it to be both publicly available and scrutinized in the details before publication. There are some minor typographic concerns as well as a general need to define terms and acronyms used throughout the manuscript (at least a supplement or in first use – see GCS for example). More substantively, there are two general issues and then some more specific minor issues of note related to the statistical results presented that listed below.

First, there are many p-values reported (at least 70 unique tests), (Table 1: 16, Table 2: 40, Table 3: 14, Table 4 (some redundant but different tests used, 45) with no control for inflated Type I error rates when “fishing” across this many different response/predictor variables. At least some of these are spurious detections without some sort of control like Bonferroni or False Discovery Rate adjustments (at least within a table or suite of similar sort of tests). I would suggest doing this for the limitation of chance false discoveries and because it would simplify the final reporting of results, if only those that are selected to have small p-values are discussed.

We are thankful to the reviewers for highlighting this. We have now removed the p-values from the tables and only describing the data for survivors and non-survivors in Table 1 and 2 as suggested by one of the reviewers. However, if the reviewers think that p-values should be reported then we can perform the Benjamini-Hochberg correction and redo these tables and results. We have tried to reduce acronyms and explain them at their first use. However, if there are any left kindly highlight.

Related to this is the redundancy of testing for survivor/not vs each variable as a response (Chi-square/Fisher’s are non-directional but the others are not) and then repeating the same tests in the univariate logistic regression models with survivor/not as the response.

Agreed, this has been removed and inferential analysis is only performed as univariable binary logistic regression with fewer plausible predictors. Thank you for the guidance.

The summary of characteristics that Tables 1 to 3 provide is interesting, but some of those tests match the Table 4 result exactly (Gender for example with a p-value of 0.859 – also note that the reported result in the Abstract about Gender is wrong if this is the correct p-value). It is also important to remember that only when p-values are 1 is there is no evidence of a difference. For example, line 133 has “There was no difference in the arterial pH” – the p-value was 0.421 (the estimated medians and quartiles match exactly which doesn’t make sense with that p-value, so should be checked) which suggests weak evidence of a difference but not “no difference”. Now if the medians do match exactly, then this statement is true but I don’t think that was support for it.

Rightly pointed out. We have corrected the interpretation errors for p-values and have tried to limit inferential analysis largely to those variables in the data which have been identified in literature as predictors of mortality.

Finally, there is the issue of selection bias in the final reported results. If you use p-values to select your final results to discuss, you overstate precision and understate the size of your p-values. Methods for adjusting for this are complex but there is a danger to model selection using p-values and then reporting the p-values as if you knew those were the predictors you were going to look at.

The analysis has been redone, and results interpreted with caution. Model selection was based on literature and plausible predictors. Interpretation is now based on magnitude of effect rather than p-value. Thank you for highlighting

Caution should be incorporated in the limitations discussion in both being too definitive in what was identified as being the only predictors that could be important and that what was identified really are the important predictors – this was a very exploratory analysis to consider so many different predictors. Additionally, no interactions were considered, so the assumption of no interactions (or at least no interactions explored) should be noted as that could change results dramatically. I am not saying they have to be, but that is an assumption.

Agreed. We have redone the analysis and discussion has been revised. Added important limitations as advised by the reviewers. Some guidance was sought from a qualified statistician in the process.

Second, there are four serious issues in the logistic regression models that need to be addressed before this might be publishable as the results hinge on these aspects of the work.

One is that there appears either to be separation issue or something close to it in both some of the univariate logistic regression models and in the reported final model. In Stata, there is supposed to be a check for this (see <https://stats.idre.ucla.edu/other/mult-pkg/faq/general/faqwhat-is-complete-or-quasi-complete-separation-in-logisticprobit-regression-and-how-do-we-deal-with-them/> for example). But the estimates are large and the CIs are very wide, which can indicate this issue, at least in some software. Were there any warning messages/automatic modifications when running the Stata logistic regression models? If so, how was that handled? As an example of one of these that I find suspicious for this issue, the final model contained oxygen saturation with an OR CI from -100 to 300. And Figure 2 does not match the numbers reported on line 173 of 1.2 to 105.9 (but the margin labels do, so there is something wrong with the lines displayed in this plot at a minimum).

This was highly insightful for us and as a result we read up on the subject. However, we did not encounter the separation issue in our data. Since the data has been changed and analysis redone, some of the skewness was already taken care of. Stata did not give us any warnings or did any automatic modifications during the analysis. We were more careful during the re-analysis to identify any such issues. Figure 2 has been replaced by a table now.

Another issue in the multivariable logistic regression is that the model building/selection process is unclear. It appears to be a forward selection process of starting with the predictor with the smallest p-value and adding others sequentially. But no details are given on what happens to get the final model from this. I was left to assume that each predictor was tried and then not kept in the model if the p-value was not small after it was added? But after the first predictor was added, you really need to try each one again one at a time to see if its addition is warranted given that new predictor and then pick the one with the smallest p-value each time. This is especially important in the possible presence of multicollinearity of predictors that is likely here (was that checked and what sort of results did it provide?). At a minimum the steps that were taken need to be clarified. Since so much rides on what is in the final model, this process should be carefully documented. With so many predictors, it is challenging to do and report the model building process. The previous suggestion of a multiple testing correction could even mean missing important predictors in this step of the process, so the variable screening before model building might not be the best approach. And with potential separation issues, I would not necessarily recommend starting with all predictors in the model and then dropping them sequentially as important predictors can get large p-values in these situations in some software (although a step-down process could be simpler to implement than step up methods).

An additional assumption in the logistic regression models is that quantitative predictors are possible and if not, then you should note this as an assumption you might make. I could imagine there are some predictors, like pH, that having either too high or too low levels could lead to adverse outcomes. Visualization of the survival response versus the quantitative predictors can help with this (and with the potential for separation issues too) and identify variables that might be candidates for polynomial treatment in the models.

The model building process has been explained in more detail in the manuscript. For the reviewers knowledge, for univariable binary logistic regression we checked linearity assumption for the continuous variables and used a p<0.25 for eligibility to the final model building. Purposeful selection was used to build the model. <https://scfbm.biomedcentral.com/articles/10.1186/1751-0473-3-17> & <https://www.ncbi.nlm.nih.gov/pmc/articles/PMC4828741/>. We used likelihood ratio test for selection of models and a change of 15% in estimates to classify for confounding. We checked for Multicollinearity using VIF criteria. We performed model goodness of fit with AUC and classification tables which have been reported in the results with the final model. I hope we will be able to satisfy the respected reviewers.

Finally, an issue with all of the logistic regression models is that you have multiple cohorts of subjects being combined. You show this in Figure 1 with the 4 months of subjects and discuss it on lines 188 to 193 which discuss changing standards of care. At least controlling for month as a categorical variable or random effect in all the logistic regression models would help to address this as a potential source of the differences in the survival outcome. With more recent focus on different variants of COVID, is it possible that this played some role in changing survival rates over time (if they were present)? The month variable would likely best not be subject to model refinement but serve as a control variable for considering all the other results.

Thank you for suggesting this. We have added a month variable in the final model to adjust for its effect.

More minor but important issues:
I am checking the box that data will be available as the authors promise that. Some Plos One authors confuse sharing summary information with sharing the entire data set as used in the models. I am assuming that is the case here. I think this is critical as others work to create meta-analysis of studies like these to synthesize local study results such as this and also because different modeling choices here could lead to very different conclusions.

No, we understand that raw data will need to be shared however, that can be done once the article is accepted for publication. Thanks

Abstract should be changed if any of the reported results are modified, which I think likely should happen. Yes it has been revised

Line 83: Did the patients admitted for other reasons have COVID too? It was unclear from this sentence. Yes, but in the re analysis they have been excluded because they had mild disease. And since the paper focuses on hospitalized COVID patients, its assumed that they are more than mild disease. Moreover, their clinical characteristics and lab parameters were different from the rest of the group.

Line 102: Why were the less than 15 year olds dropped? This relates to the limitations on line 257 and should be part of that discussion that these results are limited to subjects over 15.

15 was actually a typographical error. It was meant to be 18. The inclusion criteria was set at 18 years and above however, some patient’s data was captured who were less than 18 so removed from the analysis.

Line 89: How did you decide between t-test and Mann-Whitney U? Between the parametric Chi-squared test and Fisher’s Exact test?

Based on normality of data for t-test and Mann Whitney. Based on expected cell count of >5 in each cell for Chi-square. These tests are now not used since p-values are not reported in Table 1, 2 and 3.

Table 1: It would great to add some details on each test. A column for the numerical value of the test statistic and another for its distribution (Chi-square (df) or t(df) or “exact”/permutation for the other two versions). This adds quite a bit to the table but can help the reader fully understand what you did. There are some other issues in Table 1: First, the Age^^ suggests it should be mean +- SD but those appear to be medians (Q1 – Q3). Second is how the median results are presented – you are not reporting the IQR, you are reporting Q1 and Q3 with a minus sign between them that is confusing. I would suggest either switching to reporting the IQR = Q3-Q1 or use a comma between them. For Symptoms and Comorbid conditions – are these categories really mutually exclusive? Maybe “Others” means more than one and just wasn’t labelled well – or is it really “Other” things no in the list. I would suggest turning this into a suite of binary variables for each aspect, such as “Fever” or “No Fever” and then each can be explored for relationships to survived/not. Some may not be suitable for use in the tests (like Runny Nose that occurred a total of 5 times or Liver disease that occurred 1 time), but those rare conditions are likely going to cause you issues in the current version with small expected cell counts that violate the assumptions of the parametric Chi-squared test that was used at least for Symptoms. Table 2’s “CXR findings” and Table 3’s “In Hospital complications” also seem like they might not be mutually exclusive categories? After turning these variables into multiple binary variables (possibly), some might be useful in the logistic regression models/persist through the model refinement stages?

We are thankful to the reviewers for their suggestion. Since most of the inferential analysis has been limited to some plausible variables, most of the suggestion are unrelated to the new results. Variables like symptoms and hospital complications were not mutually exclusive but infact multiple response possible. Those in the “others” category are not mentioned in the existing categories.

Line 136: The directional interpretations (higher/lower) sound like you did one-sided tests. Is that true? If not, then try to report the p-values first and then discuss direction of differences to avoid this confusion.

The analysis has been removed

Line 160: It might be easier to discuss the results if you organize the results by groups – like for categorical predictors separate from quantitative ones? And where possible use the units of the predictors for quantitative variables and have “higher odds” not “high odds”. Maybe even a plot of these results sorted by p-values like in Figure 2 could aid the discussion of them?

The figure 2 has been removed as one of the reviewers advised against it, hence no attempt was made to depict these inferences as a figure. Most of the redundant exploratory variables have been removed from inferential analysis. We hope it is easier to understand the results now.

Table 4: As noted above, some of these are redundant with previous tests or test a similar null hypothesis (variable vs survived/not) but use a different method. It seems to make some of the earlier work redundant if this is what you really care about – and it seems like this is what you care about most. For oxygen saturation, why was the binary version with a cutoff at 93% used instead the quantitative version of it (both were used before). For the multi-category (analysis of deviance?) tests for things like “Use of Tocilizumab”, some formatting change is needed as the IVIG row looks like part of the previous multi-group test. I am assuming most are from z-tests but some are from Chi-squared tests and all are conditional on all other aspects of the models? Again a little detail on the results can help the reader understand what you did to get those.

Most of the categorical variables were defined based on clinically relevant or literature driven cut-offs rather than distribution dependant cut-offs. The chi squares have been removed now

Lines 170-175: I think this needs to be expanded. These are your main results. And it is important if your final model contains a suite of predictors to note that these results are conditional/controlled for other variables in the model – as results are different when other variables are not present. And see previous notes on the multivariable logistic regression. Adding visualizations of survived/not versus each of the final model chosen predictors could help the reader understand these results. Final model has been described and interpreted in the abstract as well as results section and discussed in the discussion too.

Line 265: I think a citation to the software used should be included. Would you be posting your code for the analyses or just the data set? Reproducible research methods that provide code run with output would help readers with other questions about results have access to that although it is not a journal requirement. We aim to provide our data to the journal. We have added citations for stata and redcap

General note: I am not sure on PlosOne’s standing on this, but Oxford commas would help in the many lists as some sentences use more complex constructions that get confusing by this absence when it could be used. noted

Figure 1: The “Referred Out” is the sample analyzed except for the under 15 year olds? How many were in the different months? This would get clarified if month of initial hospitalization were used as a predictor. We have adjusted the analysis by month now.

There are some minor typographic issues that could be resolved in another round of edits to improve readability and grammatical correctness in a few spots but it is not a poorly written paper. Noted

6. PLOS authors have the option to publish the peer review history of their article ([what does this mean?](https://journals.plos.org/plosone/s/editorial-and-peer-review-process#loc-peer-review-history)). If published, this will include your full peer review and any attached files.


If you choose “no”, your identity will remain anonymous but your review may still be made public.


**Do you want your identity to be public for this peer review?** For information about this choice, including consent withdrawal, please see our [Privacy Policy](https://www.plos.org/privacy-policy).

Reviewer #1: No

Reviewer #2: **Yes:** Christen M Gray

Reviewer #3: **Yes:** Mark C Greenwood
