# Supplementary material for: Determinants of in-hospital mortality in COVID-19; a prospective cohort study from Pakistan
Source: PLoS One. 2021 May 27;16(5):e0251754. doi: 10.1371/journal.pone.0251754 (PMC8158897; doi:10.1371/journal.pone.0251754)
Supplement: Attachment — Submitted filename: Response to reviewers.docx [file pone.0251754.s002.docx]

Dear Editor and respected reviewers,

PLOS ONE,

I am writing to you with reference to our revision titled “Determinants of in-hospital mortality in COVID-19; a prospective cohort study from Pakistan” (PNOE-D-21-00091). On behalf of my co-authors, I would like to thank the journal and respected reviewers again for their time and effort in reviewing the amendments to the paper. We are happy to know that the reviewers have acknowledged the efforts put in by the team and believe that the paper conforms to the suggested modifications.

Minor changes have been further made now as suggested by the respected reviewers. We hope to satisfy the reviewers completely this time and look forward to a quick further processing as we have now entered the 3^rd^ wave of the pandemic and this data is pending publication. Moreover, as the reviewers have not highlighted any major changes in the paper and have answered in affirmative regarding the initial screening questions from the editor, as below, we are quite hopeful that the respected editor will consider the paper for publication.

Thank you very much again for your time.

Dr Quratulain Shaikh

Corresponding author

Reviewer's Responses to Questions

**Comments to the Author**

1. If the authors have adequately addressed your comments raised in a previous round of review and you feel that this manuscript is now acceptable for publication, you may indicate that here to bypass the “Comments to the Author” section, enter your conflict of interest statement in the “Confidential to Editor” section, and submit your "Accept" recommendation.

Reviewer #1: All comments have been addressed

Reviewer #3: (No Response)

2. Is the manuscript technically sound, and do the data support the conclusions?

The manuscript must describe a technically sound piece of scientific research with data that supports the conclusions. Experiments must have been conducted rigorously, with appropriate controls, replication, and sample sizes. The conclusions must be drawn appropriately based on the data presented.

Reviewer #1: Yes

Reviewer #3: Yes

3. Has the statistical analysis been performed appropriately and rigorously?

Reviewer #1: Yes

Reviewer #3: Yes

4. Have the authors made all data underlying the findings in their manuscript fully available?

The [PLOS Data policy](http://www.plosone.org/static/policies.action#sharing) requires authors to make all data underlying the findings described in their manuscript fully available without restriction, with rare exception (please refer to the Data Availability Statement in the manuscript PDF file). The data should be provided as part of the manuscript or its supporting information, or deposited to a public repository. For example, in addition to summary statistics, the data points behind means, medians and variance measures should be available. If there are restrictions on publicly sharing data—e.g. participant privacy or use of data from a third party—those must be specified.

Reviewer #1: No

Reviewer #3: Yes

5. Is the manuscript presented in an intelligible fashion and written in standard English?

PLOS ONE does not copyedit accepted manuscripts, so the language in submitted articles must be clear, correct, and unambiguous. Any typographical or grammatical errors should be corrected at revision, so please note any specific errors here.

Reviewer #1: Yes

Reviewer #3: Yes

6. Review Comments to the Author

Please use the space provided to explain your answers to the questions above. You may also include additional comments for the author, including concerns about dual publication, research ethics, or publication ethics. (Please upload your review as an attachment if it exceeds 20,000 characters)

Reviewer #1: l. 28: " A prospective cohort study was conducted [...] to describe the predictors of mortality among hospitalized COVID-19 patients."

"describe" is a really vague objective but using the word predictor suggests that the paper is about P(survival given age).

Has been removed. This is not what we intended. Below is true

but at l. 30 "Most non-survivors were above 60 years of age (64%)"
this is about P(Age given survival)

This ambiguity should be resolved.

It has been reworded

The method section concerning the multivariate logistic regression could provide more details, in particular regarding the set of candidate explanatory variables.

We have added the details

It is claimed that "all data are fully available without restriction" but the link provided to access the data does not work.

We have uploaded the data, the link will be made public once the article is approved

Reviewer #3: Thank you for working to address reviewer comments. It is better but still needs some work to document what was done and why with the variables used and statistical modeling, and the writing is a bit rough in places.

For the response related to Table 4, “Moreover, Linearity assumptions were confirmed for continuous variables and those not fulfilling the assumption were not considered for univariable regression.”
• I did not intend for you to abandon useful predictors because they might show nonlinear relationships. There are options for transformations or polynomials to help with these situations. The papers you reference discuss using plots to check this for at least one way to qualitatively assess for clear missed curvature. This can not confirm no violation of the linearity assumption but can fail to show a problem needs to be addressed.

Thank you for guiding, as clinician readers have difficulty in comprehending transformed variables and their utility in patient care setups is limited, hence little attempt was made to transform them.

Potential bias in referrals: “However, the referral was at random depending only on bed availability.”
• This sounds more like haphazard assignment if it is related to openings in beds rather than using a truly random mechanism, like a coin flip or randomization software. It still could have bias because of this. This wording is not used in the paper.

No the referrals as mentioned many times before were on the basis of bed availability. As explained in methods section, if bed was available a patent was admitted and if not they were referred out to another facility in the city. We believe this was a global health crises and many developed nations have faced this situation where bed were not available hence it is not unique to our setting. Maybe the word random misguided the reviewer. But it was only used to explain. We have not used the word “random” in the manuscript

For the revised model selection methods, I like that you have refined this process. I don’t see information on the steps taken (what was in the model to start and what were the removal steps and those p-values – based on the papers (not cited –see below) I suspect they are bigger than 0.25 but the information on the intermediate steps is part of the evidence story) and those should be reported, not just the final model.

We are quoting an article which explains the process as “The purposeful selection process begins by a univariate analysis of each variable. Any variable having a significant univariate test at some arbitrary level is selected as a candidate for the multivariate analysis. We base this on the Wald test from logistic regression and p-value cut-off point of 0.25. More traditional levels such as 0.05 can fail in identifying variables known to be important. In the iterative process of variable selection, covariates are removed from the model if they are non-significant and not a confounder. Significance is evaluated at the 0.1 alpha level……………... A change in a parameter estimate above the specified level indicates that the excluded variable was important in the sense of providing a needed adjustment for one or more of the variables remaining in the model. At the end of this iterative process of deleting, refitting, and verifying, the model contains significant covariates and confounders. At this point any variable not selected for the original multivariate model is added back one at a time, with significant covariates and confounders retained earlier. This step can be helpful in identifying variables that, by themselves, are not significantly related to the outcome but make an important contribution in the presence of other variables. Any that are significant at the 0.1 or 0.15 level are put in the model, and the model is iteratively reduced as before but only for the variables that were additionally added. At the end of this final step, the analyst is left with the preliminary main effects model. For more details on the purposeful selection process, refer to Hosmer and Lemeshow. (1)

A crtieria of <0.10 was used as an entry criterion for the multivariable model if that is what the reviewers want to know.

There is no clear reason for many of the binary splits in predictor variables used – over 60 years? Over 93% oxygen, Procalcitonin over 2 ng/ml? Were these selected to optimally relate to the response or are the standard cutoffs or were they arbitrarily chosen? It seems like each needs a quick reason for the splits or the variables could have just been used as quantitative predictors (with linearity checks) – is that why they were split because of nonlinearity seen in the relationship to the empirical logits? Or are these standard splits? Or were they are the median or mean of the predictors? This should be explained in the article.

The categorization of variables was selected based on available emerging literature were found to be associated with mortality (1-3) (4, 5),

More specific feedback on new version:

You should decide where you are placing references, before or after periods (I think before is best) and be consistent. It is inconsistent throughout the manuscript.

Done. Thanks

Abstract:

There is a missing a space before “Most”. Extra parenthesis before 21.8. I am not sure what the group is that is being referred to in the vs 7.8% - was that survivors that had 7.8%?

Elaborated

This sentence is in need of at least some punctuation to be more clear: “…, Neutrophil to lymphocyte ratio above 5, procalcitonin above 2ng/ml, unit increase in SOFA score and arterial lactate levels while unit decrease in Pao2/FiO2 ratio and serum albumin were associated with mortality in our patients.”

Broken up into two.

Data availability:

There is a link posted but it is not active. It looks like this is just waiting until the publication is accepted to become active or the link posted was in error. I appreciate the move to archiving and posting the data set and assume this will get sorted out if the paper is accepted. I am clicking that the data are available under this understanding of the posted inactive link.

Yes, you are right. The data has been uploaded but the link will become active once made public as soon as the manuscript gets accepted

Line 69: Change “Till date” to “To this date” or “Up to the time of writing” Done

Line 71: …, these data or …, these results or …, this information. But data should be a plural word (Plos one may have a policy on this). Done

Line 90: Data were… Done

Line 94: I had not run into the term “Purposeful selection…” so you need to cite the two papers here that you are using for the model selection template that are discussed in the response to reviewers. And maybe change “advanced” to “multivariable”. And “likelihood ratio test was” to “likelihood ratio tests were”. You should document the general steps you took to implement this method – it is not a well-enough known process and some of the aspects are arbitrary choices (what p-value cut-off and how the variables are considered for re-entry) and so should be documented for the reader. And here or later on you should note that no interactions among predictors were considered and results might be very different if they were included in the model refinement process.

Added the details and reference as suggested

Line 103: The rest of … and no comma after but and cut “while” and inavailability not in availability? Done

Line 114: Similar gender distributions were or A similar gender distribution was… in both groups in Table 1 Done

Line 119: First mention of “SOFA score” – no definition or citation for it. It and any other acronyms need to be defined on first use with a citation for its source/definition at a minimum if not explained in the text. Done

Table 1 and 2: Technically the IQR is the result of calculating Q3-Q1 and a single number. I am assuming you are presenting the (first quartile – third quartile) and the table labels at least need to document that or you can actually report the IQR. Done

Line 133: capitalize Table Done

Table 3: There is a ^ symbol but I am not sure what it means as it seems to not be defined?

Table 4: For the Oxygen saturation >93%, a 1 is needed for the Crude OR to be consistent with other categorical predictor presentations.Done

Why is NLR in parentheses and what is it – it is first mentioned on line 143 and not defined. It was defined. Have marked it with a comment to highlight.

Similarly for CURB 65 and MuLBSTA? Based on the text, it seems like you only tested these predictors versus the response – so you never explored any of the ones? Or you did but are not reporting them? The evidence weakens if you are reporting selected results here. You should document how you chose to report just these predictors.

We reported univariable results in our last draft against all predictors tested however, reviewers suggested to remove unnecessary variables as the details were too distracting for the reader. Hence we only reported the ones which were selected for final model building based on cumulative evidence and abundance of reports in literature. Acronyms have been explained but these are quite standard ones which the audience must be quite familiar with. Most of the articles using these did not explain them hence we chose not to. Like for CURB-65, it actually stands for the variables included in the score- there is no expanded form and standard is to call it CURB-65. Any clinician should be familiar with the acronym. Same is the case with Mulbsta- rest of the acronyms have been explained

Line 147: you never defined which predictors were included in the full model – was it all of the Table 4 ones or did you drop gender since it was the only one with a larger p-value reported from the univariate work? Or was it all of the variables discussed in Tables 1, 2, and 3? You should report what what in the initial model and (maybe not required but it would be nice) the order of the variables dropped and possibly re-added in the “purposive” selection process from the papers you mentioned.

Since there are at least 12 variables in Table 4, the iterative process cannot be fully explained in the paper. Also it will distract the clinicians from the main purpose of the manuscript and most are not interested in statistical details. For the reviewers reference, papers defining the process have been shared and we hope they will be satisfied.

Also, you note that month was included – but was it used as a quantitative or categorical predictor – and did you see differences based on it too? I am assuming you kept it in all models regardless of its p-value, but making that clear is still needed and a reader might still be interested in changes in survival rates across months. So maybe start with including month in Table 1, 2, or 3 and then in a univariate model in Table 4 and again in Table 5. And if you clarify this early on, you do not need Line 153 with “Final model was adjusted…” – if you keep that, it should be “The final model…” or “All multivariable models were adjusted…”.

By saying that the final model was adjusted, we mean that month did not prove to be significant but still was used to adjust the effect. Upon the reviewers suggestions we have added month as a variable in the preceeding tables too. We have added the descriptives for Month variable in table 1, however the results were insignificant hence did not include in univariate results.

Line 154: I don’t usually think of using ROCs to get sensitivity and specificity – that is only one place in ROC at a cutoff of 0.5 for assigning as success/failure. But you can keep this wording if you feel strongly about it. We would want to keep it. Thanks

Table 5: Include the p-values for these terms? The purposive selection should mean all are less than 0.25 but you should still report the p-values that led to their retention in the model. It is not clear which p-values are referred to here.

These p-values refer to individual variable’s p-values in the final model. Not the ones from model selection.

Line 181: Another acronym not defined (LMIC); Line 187: What is CRS and how is it defined?; Line 198: ARDS?. added

Line 200: MODS was defined and that is helpful.- Thanks

Line 201: Maybe a caveat should be added to these two results to note that they did not end up in the final model. So something like: …, but these predictors were not retained in our final model. And then something to compare what was different about your study/methods than in citation (25) that might have led to different results on these predictors? added

Line 207: I know it is the Food and Drug Administration here, but maybe the U.S. FDA? Corrected. thanks

Line 212: HCQ is Hydroxychloroquine? Was defined in the table and written in complete form the first time Line 209. However, added HCQ with it.

Not defined again. QTc? This is the standard way of writing it- there is no elaborate form. It means corrected QT interval on ECG. And many more than follow – I’ll let the authors carefully check the rest of these.

Line 230: Is this a new paragraph? It is a very long paragraph if not. Maybe think about structure of all these results? Start with reviewing the three predictors retained and then revisit some other intriguing but ultimately abandoned predictors from the univariate results? Done

Line 259: We plan to add more subjects in the future… or We plan to add more observations in the future… Done

Line 269: Missing bracket in citation. Done

Line 279: No details on citation 8 are provided other than year and title. In my version of the manuscript, this is not the case- the full citation is there. Can you clarify or share the citation

Please check all other citations carefully as it seems many are missing critical details. For example, I also saw that line 363 had no details on citation 41 other than year and title. In our version citation 40 and 41 are used together and the reference is complete. Could you please quote the exact line as our line numbers are somehow different from the ones you are quoting.

References

1.	Bursac Z, Gauss CH, Williams DK, Hosmer DW. Purposeful selection of variables in logistic regression. Source code for biology and medicine. 2008;3(1):1-8.

2.	Caramelo F, Ferreira N, Oliveiros B. Estimation of risk factors for COVID-19 mortality-preliminary results. MedRxiv. 2020.

3.	Bonanad C, Garcia-Blas S, Tarazona-Santabalbina F, Sanchis J, Bertomeu-González V, Facila L, et al. The effect of age on mortality in patients with COVID-19: a meta-analysis with 611,583 subjects. Journal of the American Medical Directors Association. 2020;21(7):915-8.

4.	Wang X, Fang J, Zhu Y, Chen L, Ding F, Zhou R, et al. Clinical characteristics of non-critically ill patients with novel coronavirus infection (COVID-19) in a Fangcang Hospital. Clinical Microbiology and Infection. 2020;26(8):1063-8.

5.	Liu X-h, Lu S-h, Chen J, Xia L, Yang Z-g, Charles S, et al. Clinical characteristics of foreign-imported COVID-19 cases in Shanghai, China. Emerging microbes & infections. 2020;9(1):1230-2.
